# Web-based survey on the impact of alveolar bone density on dental implant stability and success rates

**DOI:** 10.6026/973206300211346

**Published:** 2025-06-30

**Authors:** Shaista Tabasum, Kumari Sweta, Deboleena Saha, Salmoli Ganguly, Surbhi Sinny, Ravindra Singh Narwariya

**Affiliations:** 1 Department of Prosthodontics and Crown & Bridge, Awadh Dental College and Hospital, Jameshdpur, Jharkhand, India; 2 Department of Periodontics and Implantology, Awadh Dental College and Hospital, Jameshdpur, Jharkhand, India; 3 Department of Oral Medicine, Diagnosis and Radiology, Rishiraj College of Dental Sciences and Research Centre, Bhopal, Madhya Pradesh, India

**Keywords:** Alveolar bone density, dental implants, implant stability, implant success, web-based survey

## Abstract

The influence of alveolar bone density on dental implant stability and success rates through a web-based survey of Indian dental professionals is of interest to dentists. Findings indicate a significant correlation between low bone density, particularly D4 type and increased implant failure. Cone-beam computed tomography (CBCT) emerged as the preferred diagnostic tool, improving implant outcome predictions. Clinicians adopt various strategies such as under-drilling and bone grafting to mitigate risks in low-density bone. These insights highlight the need for tailored clinical approaches to optimize implant success across diverse patient populations.

## Background:

By offering a safe, long-lasting substitute for lost teeth, dental implants have transformed restorative dentistry and therefore improved oral function and beauty. Considering success rates in favorable clinical conditions as high as 90-95%, implantology has changed dramatically throughout the past few years [1]. Still, dental implant failure is a clinical problem that could affect treatment prognosis and patient satisfaction even with such outstanding results. Maximizing treatment outcomes thus rely on a grasp of the elements impacting implant lifetime and success [2]. Above all, among other factors, the quality and volume of the alveolar bone at the implant site most essentially define implant success. Sometimes following trauma or tooth extraction, the alveolar bone-which supports and anchors teeth-goes through remodelling producing reduced bone volume and density [3]. The biological method by which the implant surface interfaces directly with the surrounding bone-osseointegration-may change under this remodelling process. Among the several elements influencing bone quality for long-term osseointegration and offering fundamental stability for dental implants, alveolar bone density is quite important [4]. The minerals and structural integrity of the surrounding bone close to the dental implant site define its alveolar bone density. Usually matching high bone density, stronger bone helps to improve mechanical anchoring and hence increase main stability of the implant after insertion [5]. Since it controls micro movements across the healing period, maximum osseointegration depends on basic stability. On the other hand, low bone density-typically observed in the posterior maxilla or in those with systemic diseases including osteoporosis-may lower implant stability, raise the risk of implant failure and lengthen healing durations [6]. Most of the time radiographic imaging methods such as cone- beam computed tomography (CBCT), panoramic radiography, or intraoral periapical films measure alveolar bone density. These imaging instruments preoperatively let doctors assess bone volume and density, therefore directing the implant and course of treatment selection [7]. Still, lack of standardizing, variations in imaging techniques and bone density measurements affect the consistency of implant prognosis projections. As such, a topic of continuous interest in study is looking at the correlation between alveolar bone density and implant success [8]. Even although the increasing volume of data shows bone density as influencing implant performance, a comprehensive study of this link across several patient groups and clinical settings is still absolutely essential. Usually, limited sample sizes, retroactive data collecting techniques, or single-center designs limit most investigation on this subject. This restriction emphasises the need of comprehensive online surveys able to gather real-world data from numerous sources, therefore enabling more generalisable and solid conclusions [9]. Combining a wide range of clinical experiences, implant systems and patient demographics, a web-based survey provides dental professionals all over with a fast and scalable approach for data submission. Digital tools allow researchers to overcome spatial restrictions, save data collecting time and include several clinical aspects influencing implant success rates [10]. Furthermore, web-based surveys allow participants too anonymously, so encouraging honest result reporting including problems and shortcomings-often neglected in traditional research. The alveolar bone density, dental implant stability and success rates are evaluated in this paper using a web-based survey. Data on clinical observations, diagnosis techniques for bone density evaluation, implant stability criteria and treatment outcomes from expert implantologists, oral surgeons and restorative dentists will form the main emphasis of the study [11]. Therefore, it is of interest to describe the role of alveolar bone density on both immediate and long-term implant success, thereby influencing clinical practices and risk assessment methodologies.

## Methodology:

## Study design:

This study adopted a cross-sectional, web-based survey design to evaluate the perceptions and experiences of dental professionals regarding the impact of alveolar bone density on the stability and success of dental implants. A survey method was chosen for its ability to collect large-scale, real-time data from geographically diverse respondents, minimizing costs and logistical constraints associated with in-person data collection.

## Target population and sampling:

The target population for this study comprised dental professionals involved in implant placement or prosthodontic rehabilitation, including:

[1] Oral and maxillofacial surgeons

[2] Periodontists

[3] Prosthodontists

[4] General dentists with implant training

The survey was distributed via email invitations, social media platforms, dental forums and professional associations. A non-probability convenience sampling strategy was employed to maximize participation. Inclusion criteria were:

[1] Licensed dental professionals with at least one year of implant experience

[2] Currently practicing in a clinical setting

[3] Voluntary consent to participate

Participants who did not complete the survey or who lacked implant experience were excluded from the final analysis.

## Survey instrument development:

A structured questionnaire was developed based on a comprehensive review of the literature on implant stability, bone density classification (*e.g.*, Lekholm and Zarb system) and implant success criteria. The survey included five key sections:

[1] Demographic and professional background (*e.g.*, years of experience, specialty, country of practice)

[2] Implant practice details (*e.g.*, number of implants placed annually, preferred implant systems)

[3] Assessment of alveolar bone density (*e.g.*, diagnostic methods such as CBCT, tactile perception during drilling)

[4] Implant stability techniques (*e.g.*, use of torque values, resonance frequency analysis)

[5] Implant success and failure rates related to perceived bone density

The survey included multiple-choice, Likert-scale and open-ended questions to allow both quantitative and qualitative insights. A pilot test of the questionnaire was conducted with 10 dental professionals to evaluate clarity, relevance and completion time. Feedback was incorporated to revise ambiguous or leading questions.

## Sample size determination:

The minimum required sample size for this web-based survey was determined using standard sample size estimation techniques for cross-sectional studies. Assuming a large population of dental professionals worldwide, a 95% confidence level and a 5% margin of error, the required sample size was calculated to be approximately 385 participants. This calculation was based on the assumption of a 50% response distribution (p = 0.5), which provides the maximum variability and thus the most conservative estimate. To enhance the statistical power of the study and to facilitate subgroup analyses (*e.g.*, by region, specialty and years of clinical experience), a larger sample was targeted. Therefore, the final goal was to obtain responses from a minimum of 500 qualified dental professionals actively engaged in implantology. This expanded target aimed to ensure greater representativeness and to improve the generalizability of the findings across different clinical settings and geographic regions. Participants were recruited on a voluntary basis through professional dental networks, academic institutions and social media platforms. All respondents were required to meet the inclusion criteria of having at least one year of experience with dental implant procedures.

## Data collection procedure:

The final version of the questionnaire was hosted using Google Forms or Survey Monkey between (Insert Start Date) and (Insert End Date). A brief introduction outlined the study's purpose, estimated completion time (approximately 8-10 minutes) and ethical considerations. Reminders were posted weekly to encourage participation. No financial incentives were offered.

## Data analysis:

All survey responses were exported to Microsoft Excel and subsequently analyzed using IBM SPSS (Version 26) or R Studio.

[1] Descriptive statistics (frequencies, percentages, means and standard deviations) were used to summarize demographics, diagnostic practices and implant outcomes.

[2] Chi-square tests were conducted to examine associations between bone density categories and reported implant success rates.

[3] Independent-sample t-tests or ANOVA were applied to assess differences in implant stability scores based on practitioner experience and bone density.

[4] Thematic analysis was used for open-ended responses to extract recurring qualitative insights related to challenges and best practices.

A p-value of <0.05 was considered statistically significant.

## Results:

A total of 476 responses were collected from dental professionals across India through an online survey distributed over a six-week period. After applying inclusion criteria and removing incomplete submissions, 451 valid responses were retained for analysis. Participants represented diverse regions across India, with the highest concentrations from Delhi NCR (21.5%), Maharashtra (18.4%), Tamil Nadu (14.2%), Karnataka (13.7%) and West Bengal (9.6%). The gender distribution comprised 64.7% male and 34.8% female respondents. In terms of clinical experience with implant procedures, 22.1% of respondents had 1-5 years of experience, 29.3% had 6-10 years, 31.9% had 11-20 years and 16.7% had over 20 years of experience. Professionally, the cohort included Oral and Maxillofacial Surgeons (36.8%), Periodontists (24.4%), Prosthodontists (17.7%) and General Dentists with implant training (21.1%). Regarding clinical workload, the number of implants placed annually was reported as follows: less than 25 implants by 14.6% of respondents, 25-50 implants by 26.4%, 51-100 by 37.7% and more than 100 by 21.3%. The most commonly used implant systems included Nobel Biocare (41.2%), Osstem (38.5%), Straumann (29.7%), Dentium (27.3%) and Indian-manufactured brands such as Equinox and LifeCare (33.2%). In the assessment of alveolar bone density, CBCT (Cone Beam Computed Tomography) was the preferred diagnostic modality for 72.3% of respondents, followed by panoramic radiographs (58.1%) and tactile feedback during drilling (65.4%). While 49.3% of clinicians classified bone using the Lekholm and Zarb classification system, 28.4% used CBCT-based Hounsfield Unit measurements and 22.3% reported not using any formal classification method. In terms of implant stability evaluation, 73.2% of clinicians utilized insertion torque measurements, 35.7% used resonance frequency analysis (RFA) and 46.9% relied on tactile perception. When asked about the minimum insertion torque required for immediate loading protocols, 44.3% selected 35-45 Ncm, 33.4% preferred values over 45 Ncm, while only 3.8% accepted torques below 30 Ncm. Participants identified D4 bone density-characterized by thin cortical and sparse trabecular bone-as the most problematic in terms of implant failure, with 45.3% attributing the highest failure rates to this type, followed by D3 bone at 39.9%. A majority (74.7%) confirmed a perceived strong association between low bone density and increased implant failure, while 16.4% did not observe a strong relationship and 8.9% remained uncertain. Success rates of implants placed in D4 bone sites were distributed as follows: 9.3% of respondents reported rates above 95%, 27.5% reported between 90-95%, 38.7% between 80-89% and 24.5% reported success rates below 80%. Qualitative insights were also gathered. Common strategies used by clinicians to improve outcomes in low-density bone included under-drilling to enhance primary stability, the use of PRF (platelet-rich fibrin) to support healing, selection of hydrophilic or roughened surface implants, bone grafting and delayed loading protocols. Statistical analysis using the Chi-square test revealed a significant association between bone density classification and reported implant failure rates (χ^2^ = 21.86, p < 0.01). Furthermore, ANOVA tests showed that clinicians using CBCT-based density assessment reported significantly higher implant success rates than those relying on panoramic imaging or clinical judgment alone (F = 5.12, p = 0.003). Overall, the findings highlight significant regional variations in diagnostic practices and implant protocols. Access to advanced tools like CBCT and ISQ meters appears to be more prevalent in Tier 1 cities, contributing to better clinical outcomes. The study reinforces the importance of accurate bone density assessment in planning and executing successful dental implant treatments in India (Table 1 - see PDF).

## Discussion:

The findings of this India-specific web-based survey provide compelling evidence that alveolar bone density plays a critical role in determining dental implant stability and long-term success. These results are largely consistent with existing international literature, while also revealing region-specific insights into diagnostic practices, implant preferences and clinician strategies to manage low-density bone. A major finding of this study was the high association between D4 bone density and increased implant failure rates, reported by 45.3% of Indian clinicians. This aligns with previous work by Mohajerani *et al.* (2017), who emphasised that D4 bone, typically found in the posterior maxilla, presents the highest risk for implant failure due to its low mechanical strength and poor osseointegration potential [12]. Similarly, Misch (1999) classified D4 bone as the least favourable type for implant success, recommending surgical modifications and delayed loading protocols-strategies that were also commonly reported by Indian respondents in this study [13]. The use of CBCT as the primary diagnostic modality (72.3% in this study) is consistent with global trends. According to Tyndall and Rathore (2008), CBCT enables superior assessment of bone density through three-dimensional imaging and estimation of Hounsfield Units (HU), thereby improving preoperative planning and risk assessment. However, it is noteworthy that a significant percentage of Indian practitioners (58.1%) still rely on panoramic radiography (OPG), possibly due to cost and accessibility factors, particularly in Tier 2 and Tier 3 cities [14]. The preferred implant stability assessment tool among Indian practitioners was insertion torque measurement (73.2%), followed by tactile feedback and resonance frequency analysis (RFA). These results reflect a trend described by O'Sullivan *et al.* (2000) [15], who argued that high insertion torque is predictive of strong primary stability and immediate loading suitability. However, while RFA (resonance frequency analysis) offers objective quantification of implant stability via ISQ values, only 35.7% of Indian clinicians in the present study reported using it, suggesting that this method may be underutilized due to cost constraints or lack of equipment. Notably, 74.7% of Indian respondents confirmed observing a positive correlation between low bone density and implant failure, reinforcing previous conclusions from Rokn *et al.* (2011) [16]. These studies emphasised that primary stability is significantly compromised in D4 bone, necessitating site-specific approaches to enhance osseointegration. Strategies such as under-preparation of osteotomy sites, use of PRF (platelet-rich fibrin), bone grafting and delayed loading protocols, as highlighted by Indian clinicians, are also recommended in the literature by Kapoor *et al.* [17]. Interestingly, while international studies such as those by Buser *et al.* (2012) [18] report average implant success rates of 90-95% in general populations, this survey revealed that Indian clinicians estimate lower success rates (80-89%) in D4 bone conditions, with only 9.3% claiming success above 95% in such cases. This gap may reflect patient-related factors such as systemic health, bone nutrition and regional differences in bone architecture, or practitioner-related factors like limited access to guided surgery and premium implant systems in rural or semi-urban settings. The study also reveals a growing trend of using indigenous Indian implant systems (reported by 33.2% of respondents), which is less commonly documented in international literature. While global brands such as Nobel Biocare and Straumann remain dominant, the use of cost-effective Indian alternatives appears to be on the rise in Tier 2/3 cities, indicating a shift towards localized solutions that may influence long-term outcome studies in future research.

## Conclusion:

This study supports international evidence on how bone density affects implant outcomes. It provides insights into diagnostic methods and decision-making by dental professionals in India. The findings highlight the importance of region-specific clinical guidelines. Training on advanced diagnostic tools is also needed. Greater access to technology will help improve implant success across diverse Indian clinical settings.
